# α1-antitrypsin mitigates NLRP3-inflammasome activation in amyloid β_1–42_-stimulated murine astrocytes

**DOI:** 10.1186/s12974-018-1319-x

**Published:** 2018-09-27

**Authors:** Taraneh Ebrahimi, Marcus Rust, Sarah Nele Kaiser, Alexander Slowik, Cordian Beyer, Andreas Rembert Koczulla, Jörg B. Schulz, Pardes Habib, Jan Philipp Bach

**Affiliations:** 1 Department of Neurology, RWTH Aachen University, Aachen, Germany; 2 Institute of Neuroanatomy, RWTH Aachen University, Aachen, Germany; 3 Department of Internal Medicine, Pulmonary and Critical Care Medicine, University Medical Center Giessen and Marburg, Marburg, Germany; 4 JARA-Institute Molecular Neuroscience and Neuroimaging, Forschungszentrum Jülich GmbH and RWTH Aachen University, Aachen, Germany

**Keywords:** Neuroinflammation, NLRP3, NALP3, Inflammasome, Alzheimer’s disease, Amyloid β, Alpha 1-antitrypsin, Astrocytes

## Abstract

**Background:**

Neuroinflammation has an essential impact on the pathogenesis and progression of Alzheimer’s disease (AD). Mostly mediated by microglia and astrocytes, inflammatory processes lead to degeneration of neuronal cells. The NLRP3-inflammasome (NOD-like receptor family, pyrin domain containing 3) is a key component of the innate immune system and its activation results in secretion of the proinflammatory effectors interleukin-1β (IL-1β) and interleukin-18 (IL-18). Under physiological conditions, cytosolic NLRP3-inflammsome is maintained in an inactive form, not able to oligomerize. Amyloid β_1–42_ (Aβ_1–42_) triggers activation of NLRP3-inflammasome in microglia and astrocytes, inducing oligomerization and thus recruitment of proinflammatory proteases. NLRP3-inflammasome was found highly expressed in human brains diagnosed with AD. Moreover, NLRP3-deficient mice carrying mutations associated with familial AD were partially protected from deficits associated with AD.

The endogenous protease inhibitor α1-antitrypsin (A1AT) is known for its anti-inflammatory and anti-apoptotic properties and thus could serve as therapeutic agent for NLRP3-inhibition. A1AT protects neurons from glutamate-induced toxicity and reduces Aβ_1–42_-induced inflammation in microglial cells. In this study, we investigated the effect of Aβ_1–42_-induced NLRP3-inflammasome upregulation in primary murine astrocytes and its regulation by A1AT.

**Methods:**

Primary cortical astrocytes from BALB/c mice were stimulated with Aβ_1–42_ and treated with A1AT. Regulation of NLRP3-inflammasome was examined by immunocytochemistry, PCR, western blot and ELISA. Our studies included an inhibitor of NLRP3 to elucidate direct interactions between A1AT and NLRP3-inflammasome components.

**Results:**

Our study revealed that A1AT reduces Aβ_1–42_-dependent upregulation of NLRP3 at the mRNA and protein levels. Furthermore, A1AT time-dependently mitigated the expression of caspase 1 and its cleavage product IL-1β in Aβ_1–42_-stimulated astrocytes.

**Conclusion:**

We conclude that Aβ_1–42_-stimulation results in an upregulation of NLRP3, caspase 1, and its cleavage products in astrocytes. A1AT time-dependently hampers neuroinflammation by downregulation of Aβ_1–42_-mediated NLRP3-inflammasome expression and thus may serve as a pharmaceutical opportunity for the treatment of Alzheimer’s disease.

**Electronic supplementary material:**

The online version of this article (10.1186/s12974-018-1319-x) contains supplementary material, which is available to authorized users.

## Background

Alzheimer’s disease (AD) is the most common form of dementia with more than 40 million patients affected worldwide [1]. By 2050, the number is expected to quadruple [2]. Age is the most important risk factor [3], because the incidence of AD doubles every 5 years after the age of 65 years [3]. There is no causal treatment so far. To date, almost all biologicals or secretase inhibitors have failed in clinical trials which emphasize the need for further research into novel therapeutic options. Treatment of patients, even with early symptoms, only starts when the disease pathology has progressed and neural tissue has irreversibly been damaged for years. Therefore, current trials focus on patients with prodromal disease signs [4–11].

Following the amyloid hypothesis, accumulation of extracellular Aβ_1–42_-oligomers is one of the earliest and driving factors for pathogenesis of AD [12, 13]. The majority of in vitro studies investigated the effect of Aβ_1–42_ after an incubation time of 24–72 h [14–16]. Current scientific literature reveals less data about a possible damaging effect of Aβ_1–42_-stimulation on the central nervous system (CNS) after short-term stimulation of only a few hours [14].

Besides Aβ_1–42_, AD is mainly characterized by hyperphosphorylation of tau and neuroinflammation mediated by microglia and astrocytes, causing neuronal cell death [17–21]. A key component of the innate immune system is the NOD-like receptor family, pyrin domain-containing 3 (NLRP 3) [22, 23]. Though ubiquitously expressed in CNS, NLRP3 is found highly expressed in Alzheimer’s patients’ brains [22–24]. Under physiological conditions, an inactive form of NLRP3 is located in the cytoplasm [14, 25]. However, in the absence of activating signals, the NLRP3-inflammasome is not able to oligomerize [25]. After NLRP3-receptors recognize danger signals released by damaged cells and pathogens [26], NLRP3, the adaptor protein ASC (apoptosis-associated speck-like protein containing a CARD) and pro-caspase 1 form a subcellular multiprotein complex, known as NLRP3-inflammasome [22, 23, 27]. Subsequently, pro-caspase 1 is activated by autoproteolysis and catalyzes the cleavage of the precursors pro-IL-1β and pro-IL-18 [27]. Mostly induced by microglia and astrocytes, secretion of pro-inflammatory cytokines IL-1β and IL-18 drives inflammatory responses and causes neuronal damaging [27–29].

Inflammasomes are linked to neurodegenerative diseases: activated NLRP3 was observed in Parkinson’s disease in the midbrain and cerebrospinal fluid [30–33]. Furthermore, in an experimental ischemic stroke model, NLRP3-deficiency was protective against ischemic neuronal damage [34].

In a cellular model of Alzheimer’s disease, Halle et al. 2008 first described that activation of NLRP3-inflammasome is induced by Aβ_1–42_ in microglia, leading to an overexpression of the pro-inflammatory cytokine IL-1β [14]. Moreover, Aβ_1–42_ activates the NLRP3-inflammasome in astrocytes [35]. Alike microglia, as a part of the CNS immune response, reactive astrocytes surround amyloid deposits and perform phagocytosis [35–37]. Most studies investigated Aβ_1–42_-mediated inflammatory processes in microglia and little is known about inflammasome activation in Aβ_1–42_-stimulated astrocytes [14, 15, 24, 38–40]. Since inflammation occurs as one of the first cellular and molecular responses after cell stress, short-term effects of Aβ-stimulation in astrocytes need further characterization. Aside from cell culture models, also in human models of AD high expression of NLRP3-inflammasome was found. More precise, an upregulation of NLRP3 expression in peripheral monocytes from individuals with AD was identified [16]. Moreover, in frontal cortex and hippocampus lysates from AD patients increased amounts of cleaved caspase 1 were detected [24]. Interestingly, NLRP3 deficient mice carrying genes associated with familial AD were protected from spatial memory deficits [24].

Therefore, a potent inhibition of the NLRP3-inflammasome could be a new therapeutic approach. The protease inhibitor α1-antitrypsin (A1AT) is known for its anti-inflammatory and anti-apoptotic properties in both hepatic and lung cells [41–44]. Conveniently, A1AT is therapeutically used in patients with A1AT-deficiency and therefore well-established as a pharmaceutical agent. Recently, we demonstrated that A1AT also protected neurons from glutamate-induced toxicity [45] and reduced Aβ_1–42_-induced inflammation in microglial cells [15]. In addition, we found that A1AT inhibited calpain and stabilized calcium-homeostasis [15]. This study investigated the regulation of NLRP3-inflammasome by A1AT in Aβ_1–42_-stimulated murine astrocytes. In order to elucidate a direct interaction between A1AT and the NLRP3-inflammasome, we have included an inhibitor of NLRP3. MCC950 is a highly potent and specific inhibitor of NLRP3, without affecting AIM2, NLRC4, or NLRP4 [46–49]. Recent data revealed that MCC950 stimulated Aβ-phagocytosis in vitro, and reduced Aβ-accumulation in a mouse model of AD, which was associated with improved cognitive function [50].

## Methods

### Primary cortical murine astrocyte culture

Postnatal (P0 to P2) cortical astrocyte culture preparation from BALB/c mice (Charles River) was performed as previously described by Habib et al. 2014 [51]. Preparation was conducted in accordance with animal welfare policy of University Hospital Aachen and the government of the State of North Rhine-Westphalia, Germany (no. 84.02.04.2015.A292). Briefly, after brain dissection meninges and blood vessels were removed, cortex was isolated, homogenized, and dissolved in Dulbecco’s phosphate-buffered saline (DPBS, Life Technologies, USA) containing 1% (*v*/*v*) trypsin and 0.02% (*v/v*) EDTA. The cell suspension was filtered through a 50 μm nylon mesh. After centrifugation (1400 rpm, 5 min), pellets were re-suspended in Gibco™ Dulbeccos’s modified Eagle medium (DMEM, Life Technologies, USA) and seeded on flasks in DMEM with additional 10% fetal bovine serum (FBS, PAA, Austria) and 0.5% penicillin-streptomycin (Invitrogen, USA). All flasks and plates were coated by poly-L-ornithine (PLO, Sigma-Aldrich, Germany) prior to cell seeding. Cells were kept in a humidified incubator at 37 °C and 5% CO_2_. After cell confluence was about 80%, flasks were shaken for 2 h (150 rpm, 37 °C) to remove microglia and oligodendrocytes from astrocytes. Additionally, before each subcultivation the 2 h machine shaking was repeated, the contaminating cells were transferred to the medium and then removed.

**Fig. 1:**
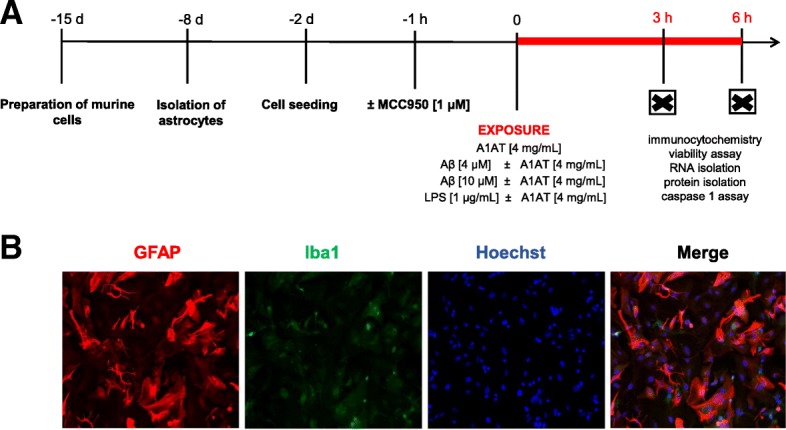
Experimental setting. **a** Cortices from BALB/c mice were prepared and seeded on flasks 2 weeks prior to stimulation. After a week, when cell confluence was about 80%, flasks were shaken to remove microglia and oligodendrocytes from astrocytes. At passage 2, astrocytes were seeded on experimental plates 48 h prior to stimulation. After short-term stimulation (3 h/ 6 h) with two concentrations of Aβ_1–42_ (4, 10 μM) or LPS (1 μg/mL) and treatment with A1AT (4 mg/mL) immunocytochemistry, viability assays, caspase 1 assay, RNA and protein isolation were performed. For our control experiments, pretreatment with MCC950 (1 μM) was performed 1 h prior to stimulation. **b** Astrocyte purity was assessed by ICC using GFAP and Iba1 combined with Hoechst for DNA staining

For subcultivation, cells were trypsinized with 2.5% (*v*/*v*) trypsin diluted in PBS/EDTA and seeded on new flasks in a 1:3 ratio. Medium was refreshed every second day. Subcultivation was performed when cells reached a confluence of about 80%. At passage 2, astrocytes were seeded on experimental plates 48 h prior to stimulation. 24 h before stimulation medium was changed to phenol red-free Gibco™ Roswell Park Memorial Institute (RPMI 1640, Life Technologies, USA) with additional 5% FBS and 0.5% penicillin-streptomycin (Fig. 1a).

Astrocyte culture purity was examined by immunocytochemistry (ICC) using anti-GFAP-antibody (glial fibrillary acidic protein), anti-Iba1-antibody (ionized calcium binding adaptor molecule 1), anti-Olig2-antibody (oligodendrocyte transcription factor 2) and Hoechst (33342, Trihydrochloride, Trihydrate, Invitrogen, USA) for nucleus staining. A detailed list of antibodies used for ICC is illustrated in Table 1. The average of astrocyte purity was 95%, less than 5% of the cells were microglia, under 0.5% of the cells remained undefined (Additional file 1: Figure S2B).

**Table 1:** Antibodies for immunocytochemistry

Antibody	Company	Order no.	Host	Dilution
Anti-goat 488	Life Technologies, USA	A11055	Donkey	1:500
Anti-goat 594	Life Technologies, USA	A11058	Donkey	1:500
Anti-mouse 488	Life Technologies, USA	A21202	Donkey	1:500
Anti-mouse 594	Life Technologies, USA	A21203	Donkey	1:500
Anti-rabbit 488	Life Technologies, USA	A21206	Donkey	1:500
Anti-rabbit 594	Life Technologies, USA	A21207	Donkey	1:500
GFAP	EnCor Biotechnology, USA	RPCA-GFAP	Rabbit	1:1000
Iba 1	Abcam, UK	ab107159	Goat	1:200
Iba 1	Millipore, USA	MABN92	Mouse	1:600
Iba 1	Wako, Japan	019–19,741	Rabbit	1:1000
NLRP3	Adipogen, USA	AG-20B-0014	Mouse	1:333
Olig2	Millipore, USA	MABN50	Mouse	1:1000
Olig2	Millipore, USA	AB9610	Rabbit	1:500

Primary and secondary antibodies used for immunocytochemical staining are listed

### Preparation of A1AT, amyloid β_1–42_, LPS, and MCC950

A1AT originated from Prolastin (Grifols, Barcelona, Spain). 1000 mg of the powder were dissolved in 25 mL ultrapure water to obtain a concentration of 40 mg/mL. The solution was aliquoted and stored at − 80 °C.

To generate Amyloid β_1–42_ oligomers, we used the procedure described by Kayed et al. [52] and Gold et al. [15]**.** Briefly, 300 μg Aβ_1–42_ (Bachem, Bubendorf, Switzerland) were dissolved in 90 μL hexafluoroisopropanol, 210 μL ultrapure water and diluted with 900 μL 100 mM NaCl, 50 mM Tris (pH 7.4). The solution was stirred for 48 h on a magnetic stirrer at room temperature. Next, the tube was weighed again, and weight difference was adjusted with 100 mM NaCl 50 mM Tris (pH 7.4). The Aβ_1–42_ concentration of this solution was 56 mM. After centrifugation (16,000×*g*, 10 min), the supernatant was used for cell culture experiments. A negative control containing all ingredients but Aβ_1–42_ was established to evaluate possible solvent effects on astrocytes. Lipopolysaccharides (LPS) from *Escherichia coli* (Sigma-Aldrich, Germany) were used at a concentration of 1 μg/mL, as an extra stimulus for maximum cell stimulation. Stimulation time of all reagents was 3 h and 6 h. In order to reveal the short-term inflammasome regulation after Aβ_1–42_ and to understand the mechanism of early inflammation in AD, we decided for short term stimulation of cells.

MCC950 (Adipogen, USA) was diluted in DMSO (dimethylsulfoxid, Sigma-Aldrich, Germany) and used in a concentration of 1 μM. MCC950 incubated 1 h before further treatment, according to previous studies by Coll et al. [46]. Then, treatment with A1AT, Amyloid β_1–42_, and LPS was performed for 3 h and 6 h.

Cells were kept in the incubator at 37 °C and 5% CO_2_.

### Dose-dependency studies

A dose-dependency study with increasing concentrations of A1AT [1, 2, 4, 8, 10, and 12 mg/mL] and Aβ_1–42_ [1, 2, 4, 8, 10, and 12 μM] was performed to determine the sublethal concentration for primary astrocytes. Lactate dehydrogenase (LDH) and Cell Titer-Blue (CTB) assay were used to assess cell viability after 3 h stimulation.

### Cell viability

#### LDH-release

CytoTox 96® Non-Radioactive Cytotoxicity Assay (Promega, USA) was performed according to the manufacturer’s protocol to measure release of lactate dehydrogenase (LDH) as a marker of cellular viability. Astrocytes were seeded on a 96-well plate 48 h prior to stimulation and were finally stimulated with Aβ_1–42_ or LPS, and treated with A1AT. After 3 h/6 h incubation time, 50 μL of each well was transferred to a fresh 96-well plate. In addition to a no-treatment-cell control, a no-cell control, one positive control containing LDH and a control containing astrocytes lysed with Triton X-100 were used. CytoTox 96® Reagent (Promega, USA) was added to each well, and the absorbance was recorded at 490 nm by Infinite® M200 (Tecan, Switzerland). Data are presented as percentage of maximum LDH release (100%), which was determined by astrocytes lysed with 1% Triton X-100.

#### CTB assay

CellTiter-Blue® Cell Viability Assay (Promega, USA) was performed according to manufacturer’s protocol to assess metabolic activity of the cells. Astrocytes were seeded on a 96-well plate 48 h prior to stimulation and finally stimulated with Aβ_1–42_ or LPS and treated with A1AT. After 3 h and 6 h incubation time, CellTiter-Blue® Reagent (Promega, USA) was added to each well. After 2.5–3 h, a color switch (reduction of resazurin) was observed and fluorescence was recorded at 560_Ex_/590_Em_ by Infinite® M200 (Tecan, Switzerland).

### Semi-quantitative and quantitative real-time PCR

After 3 h/6 h of stimulation, medium was removed and peqGOLD TriFast™ (Peqlab, Germany) was added to cells. RNA was isolated using phenol-chloroform extraction method as previously described [53]. Afterwards, RNA concentration was measured by NanoDrop® ND-1000 (Thermo Fisher Scientific, USA). RNA purity was examined using 260/280 ratio, which was at 2.0 ± 0.1. Samples were diluted with ultrapure water to attain same RNA concentration in each sample. For DNA transcription, samples were transcribed by moloney murine leukemia virus (M-MLV) reverse transcriptase (Invitrogen™, USA) using random primer (Invitrogen™, USA). Semi-quantitative PCR with 30–32 cycles was performed to assess cDNA transcription success, starting with reference genes primer HPRT (hypoxanthine phosphoribosyltransferase 1), GAPDH (glycerinaldehyd-3-phosphate-dehydrogenase), and Hsp90 (heat shock protein 90). Table 2 contains all primers used for PCR. Positive control contained mouse cortex and negative control contained ultrapure water. A Thermocycler Mastercycler ep gradient S (Eppendorf, Germany) was used with the following settings: 3 min at 95 °C, 40 s at 95 °C, 40 s at respective annealing temperature (Table 2), and 45 s at 72 °C, 45 s at 72 °C. Nucleic acids were detected after application on 3% agarose gel containing Midori Green Advance (Biozym, Germany) for DNA staining and electrophoresis (25 min, 125 V constant, 400 mA). Gels were then photographed in E-box VX2 (Peqlab, Germany).

**Table 2:** Primers for PCR

Primer	Sequence	Bp	AT
ASC	Forward: CTTGTCAGGGGATGAACTCAAAA Reverse: GCCATACGACTCCAGATAGTAGC	154	60
Casp1	Forward: CCGTGGAGAGAAACAAGGAGT Reverse: CCCCTGACAGGATGTCTCCA	180	62
GAPDH	Forward: TGTGTCCGTCGTGGATCTGA Reverse: CCTGCTTCACCACCTTCTTGA	77	65
HPRT	Forward: GCTGGTGAAAAGGACCTCT Reverse: CACAGGACTAGAACACCTGC	249	61
Hsp90	Forward: TACTACTACTCGGCTTTCCCGT Reverse: TCGAATCTTGTCCAGGGCATC	192	64
IL-1β	Forward: CAGCTCATATGGGTCCGACA Reverse: CTGTGTCTTTCCCGTGGACC	251	61
IL-18	Forward: SGCCTGTGTTCGAGGATATGACT Reverse: CCTTCACAGAGAGGGTCACAG	122	62
NLRP3	Forward: CCTGGGGGACTTTGGAATCA Reverse: GATCCTGACAACACGCGGA	113	65

List of primers, respective sequences, base pairs (bp) and annealing temperature in °C (AT) used for sq-PCR and q-RT-PCR

For quantitative real-time PCR, a dilution series containing all samples was established, starting from 100% with dilution factor 2. Then, samples were diluted 1:10 with ultrapure water. Master mixture included SensiMix™ SYBR and Fluorescein (Bioline, USA), ultrapure water and primer (Table 2). CFX Connect™ Real-Time PCR Detection System (Bio-Rad, USA) was used. The following settings were adjusted: 10 min at 95 °C, 15 s at 95 °C, 30 s at respective annealing temperature (40 cycles), 30 s at 72 °C, and 5 s at 72 °C. The software quantified DNA products by melting curve analysis. An addition gel electrophoresis was performed to control the size of the amplified DNA products. First, the expression of reference genes was measured. All following target gene expressions were normalized to reference genes HPRT, GAPDH, and Hsp90. Using the qbase+ software (Biogazelle, Belgium), the relative quantification was calculated by the ∆∆Ct-method and data were expressed as relative amount of the three housekeeping genes, respectively, by using the multiple reference gene normalization method. Untreated cell controls were set to 1.

### Immunocytochemistry

Immunocytochemistry (ICC) was performed as previously described by Habib et al. 2014 [51]. Astrocytes were seeded on cover slips on a 24-well plate. After stimulation, cells were fixed with 3.7% paraformaldehyde, lysed with Triton X-100, blocked with blocking buffer, and incubated with primary antibody. A negative control was established by incubating the cover slip only with blocking buffer without primary antibody. On the next day, the secondary antibody was applied and incubated for 2 h. After washing the cover slips, nuclei were stained by Hoechst (33342, Trihydrochloride, Trihydrate, Invitrogen, USA). A detailed list of antibodies used for ICC can be found in Table 1. Fluorescence images were taken with Leica DM6000 B (Leica Microsystems, Germany). For each experiment, the identical microscope settings were selected. Fluorescence intensity was measured using ImageJ (USA),

### Western blot

Samples were generated from cell lysate and supernatant. Pierce™ BCA Protein Assay Kit (Thermo Fisher Scientific, USA) was used according to manufacturer’s protocol to measure protein concentration. The absorbance was recorded at 562 nm by Tecan Infinite® M200 reader (Tecan, Switzerland). Western blot was performed as previously described by Dang et al. 2011 [54, 55]. Briefly, after astrocytes were stimulated for 3 h/ 6 h, the lysis and extraction buffer as well as protease inhibitors were added. The buffer consists of 10 mM HEPES (PromoCell, Germany), 1.5 mM MgCl2 (Sigma-Aldrich, Germany), 10 mM KCl, 0.5 mM DTT, and 0.05% NP-40 (pH = 7.9.).

Samples were heated for 5 min at 95 °C, loaded on gels, and electrophoresis was performed (10 min at 80 V, 1 h at 140 V). PVDF membranes (Trans-Blot® Turbo™ RTA Mini PVDF Transfer Kit, Bio-Rad, USA) were activated with methanol, and then blotting was performed by Trans-Blot® Turbo™ Transfer System (Bio-Rad, USA) (22 min, 14 V). Blotting success was verified by incubating the membrane with methanol and Ponceau S. The membrane was incubated with the primary antibody overnight; on the next day, the secondary antibody was added after washing the membrane. Table 3 reveals the antibodies used. Chemiluminescence detection system was performed using Pierce™ ECL Western Blotting Substrate (Thermo Fisher Scientific, USA). Densitometric analysis was performed using ImageJ Software (USA).

**Table 3:** Antibodies for western blotting

Antibody	Company	Order no.	Host	Dilution
Anti-mouse	Sigma-Aldrich, Germany	A4416	Goat	1:4000
Anti-rabbit	Bio-Rad, USA	170–6515	Goat	1:5000
ASC (N15)-R	Santa Cruz, USA	sc-22514-R	Rabbit	1:1000
Caspase 1	Adipogen	20B0042C100	Mouse	1:1000
Caspase 1 p10	Santa Cruz, USA	sc-514	Rabbit	1:1000
Caspase 1 p20	Bioss	bs-6368R	Rabbit	1:500
IL-1β	Abcam, UK	ab9722	Rabbit	1:1000
IL-1β	Cell Signaling Technology, USA	12242S	Mouse	1:1000
IL-18	Abcam, UK	Ab71495	Rabbit	1:1000
IL-18	Santa Cruz, USA	sc-7954	Rabbit	1:1000
NLRP3	Bioss, USA	bs-10021R	Rabbit	1:1000
β-actin	Santa Cruz, USA	sc-47,778	Mouse	1:5000

List of primary and secondary antibodies used for western blotting. Antibodies were diluted in 5% milk

### ELISA

Samples were generated from supernatant. Pierce™ BCA Protein Assay Kit (Thermo Fisher Scientific, USA) was used according to manufacturer’s protocol to measure protein concentration. The absorbance was recorded at 562 nm by Tecan Infinite® M200 reader (Tecan, Switzerland). Murine IL-1β Standard ABTS ELISA (PeproTech, USA) was performed according to manufacturer’s protocol. Capture antibody was incubated on a 96-well plate overnight. Wells were blocked for 1 h then incubated overnight with standard and samples in triplicate. Next, detection antibody was incubated for 2 h. Avidin-HRP conjugate was incubated for 30 min, afterwards ABTS was added. The color development was recorded at 405 nm by Tecan Infinite® M200 reader (Tecan, Switzerland).

### Caspase 1 assay

FAM-FLICA® Caspase-1 Assay Kit (ImmunoChemistry Technologies, USA) was performed according to the manufacturer’s protocol to detect caspase 1 activity after 3 h and 6 h stimulation time. FLICA was incubated for 1 h at 37 °C. Hoechst 33342 (1:10000) was used for nuclear staining, cells then were fixed with 3.7% paraformaldehyde. Fluorescence images were taken with Leica DM6000 B (Leica Microsystems, Germany); for each experiment, the exact same microscope settings were adjusted. The number of caspase 1 active cells was counted and set in relation to the total amount of counted astrocytes per well. For each treatment group, 100 cells/well in 5 wells were counted.

### Data analysis

All experiments were performed at least three times in triplicate. All data are presented as arithmetic mean ± standard deviation of the mean. Prior to the analysis the residuals of data were tested for normal distribution with the Shapiro-Wilk normality test using JMP® (Version 10, SAS Institute Inc., Cary, NC, USA, 1989–2007). Secondly, equal variance was tested with the Bartlett test. In case that one of these tests was significant, a Box-Cox transformation was performed, and the test for normality and equal variance were repeated with the new calculated values. Finally, a one-way ANOVA was applied followed by the Tukey-HSD post-hoc test for intergroup differences. When transformation of data failed to convert non-normal into normal distributed data, rank data were calculated and used for one-way ANOVA analysis, which results in the Kruskal-Wallis non-parametric analysis followed by the Tukey-HSD post-hoc test. Statistical significance was set at *p* value ≤ 0.05 (*^/a^ ≤ 0.05, **^/aa^ ≤ 0.01, ***^/aaa^ ≤ 0.001).

## Results

After primary astrocytes were stimulated according to work flow (Fig. 1a), culture purity of 95% astrocytes on average was examined by ICC, less than 5% of the remaining cells were microglia (Fig. 1b, Additional file 2: Figure S1 and Additional file 1: Figure S2).

### Amyloid β_1–42_ had a dose-dependent cytotoxic effect on astrocytes

First, we incubated astrocytes with Aβ_1–42_ for 3 h to assess the short-term effect on cell viability. A concentration range of frequently used doses between 1 μM and 12 μM was selected [14, 15]. Aβ_1–42_ induced a concentration-depended release of LDH into the medium, reaching 50% release and a significant difference compared to the control condition at a concentration of 12 μM (Additional file 3: Figure S3A). In comparison, the stimulation with LPS (1 μg/mL) led to a LDH-release of about 60%. The metabolic activity of Aβ_1–42_-stimulated primary astrocytes (Cell Titer-Blue-assay) revealed no significant dose-dependent differences (Additional file 4: Figure S4A). To rule out cytotoxic effects, the further studies were performed with sublethal doses of Aβ_1–42_ at 4 μM and 10 μM.

To examine a possible cytotoxic effect of A1AT, dose-dependency studies were performed. 3 h incubation of astrocytes with increasing concentrations of 1 mg/mL to 12 mg/mL of A1AT did not affect LDH release (Additional file 3: Figure S3B), also CTB assay showed no influence on metabolic activity of astrocytes (Additional file 4: Figure S4B).

**Fig. 2:**
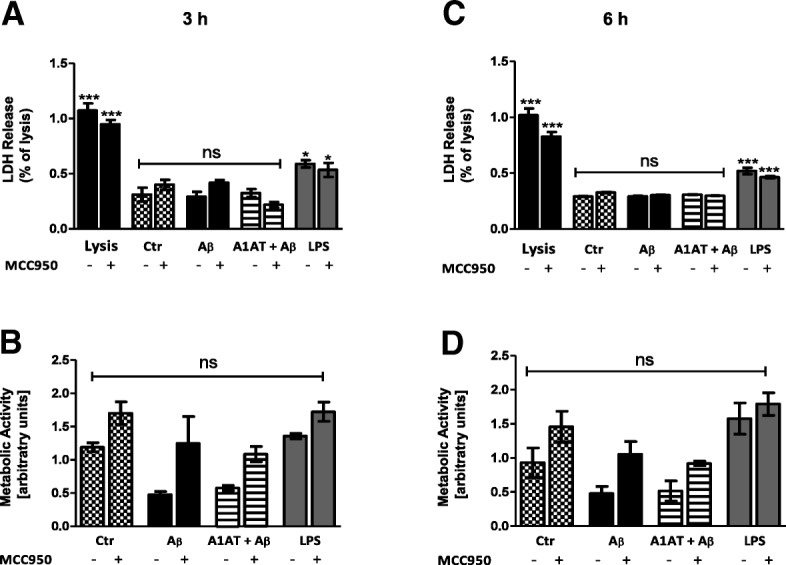
Effect of Aβ_1–42_ stimulation and A1AT treatment ± MCC950 on cell viability at 3 h and 6 h. Treatment with Aβ_1–42_ [4 μM], A1AT [4 mg/mL], and MCC950 [1 μM] did not affect LDH-release at 3 h and 6 h stimulation, whereas LPS-stimulation [1 μg/mL] significantly increased LDH-release (**a**, **c**). Cell lysis defined maximal (100%; 1.0) release of LDH. MTT-assay (**b**, **d**) revealed a trend towards decrease in cell metabolism after 3 h and 6 h Aβ_1–42_-stimulation, which was not significant. Treatment with MCC950 led to an increase of cell metabolism in each treatment group at 3 h and 6 h, which was not significant. *^/a^*p* < 0.05; **^/aa^*p* < 0.01; ^***/aaa^*p* < 0.001, ns not significant compared to untreated cell control (ctr)

Moreover, co-exposure of astrocytes with Aβ_1–42_ (4 μM and 10 μM) and A1AT (1 mg/mL) had no impact on LDH release (Fig. 2a, c, Additional file 3: Figure S3C) or cell metabolism (Fig. 2b, d, Additional file 4: Figure S4C). In contrast, A1AT exposure of LPS-stimulated astrocytes significantly reduced LDH release (Additional file 3: Figure S3C).

### A1AT prevented Aβ_1–42_-induced upregulation of NLRP3 mRNA and protein

**Fig. 3:**
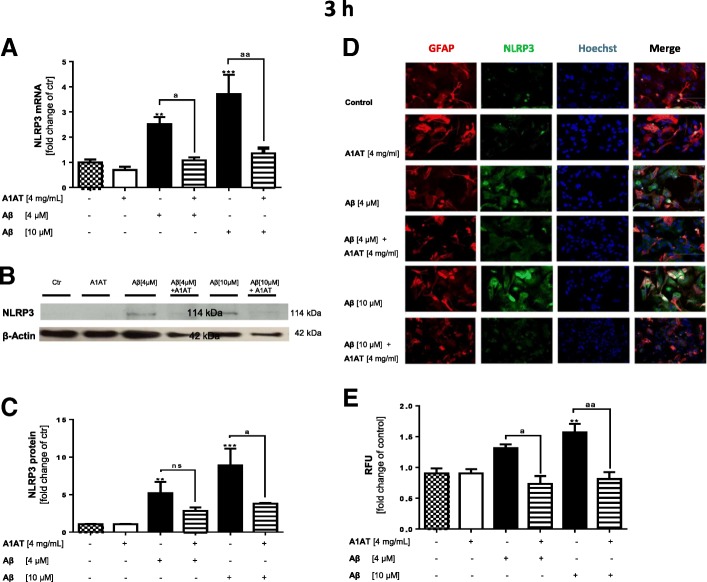
Treatment with A1AT abrogated Aβ_1–42_-induced upregulation of NLRP3 mRNA and protein in primary astrocytes. **a** As quantified by RT-PCR treatment with 4 μM and 10 μM of Aβ_1–42_ significantly increased mRNA levels of NLRP3 in astrocytes. In addition, co-treatment with 4 mg/mL of A1AT blocked this increase in NLRP3 mRNA expression significantly. Data of *n* = 6 in triplicate represent mean ± SD. **b**–**c** Densitometric analysis of western blots confirmed an Aβ_1–42_-induced significant increase of NLRP3 protein levels at 3 h. Treatment with 4 mg/mL of A1AT significantly attenuated this increase at 10 μM Aβ_1–42_. Data of *n* = 3 in triplicate represent mean ± SD. **d**–**e** Astrocytes treated with 10 μM of Aβ_1–42_ showed a significant increase of NLRP3-fluorescence intensity, whereas co-treatment with A1AT significantly reduced NLRP3-expression. Fluorescence images of each experiment were taken using the exact same microscope settings and fluorescence intensity was measured by ImageJ (USA). Data of *n* = 4 in triplicate represent mean ± SD. *^/a^*p* < 0.05; **^/aa^*p* < 0.01; ^***/aaa^*p* < 0.001, ns not significant compared to untreated cell control (ctr)

**Fig. 4:**
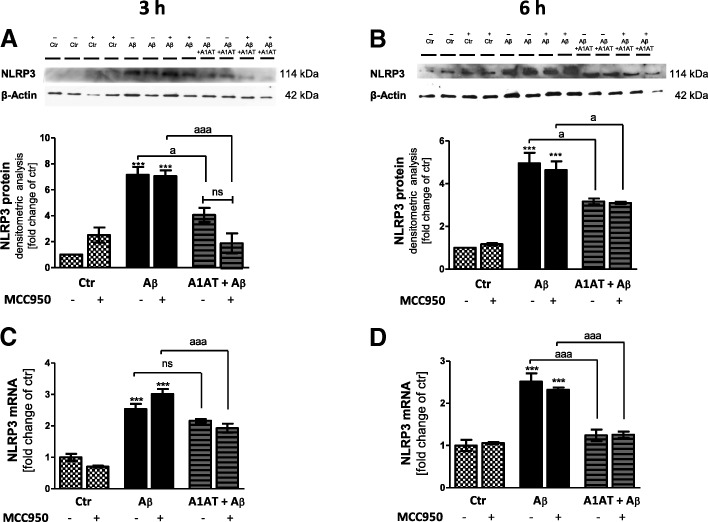
Treatment with A1AT abrogated Aβ_1–42_-induced upregulation of NLRP3 mRNA and protein in primary astrocytes. Western blot confirmed an Aβ_1–42_-induced significant increase of NLRP3 protein levels at 3 h (**a**) and 6 h (**b**). Co-treatment with A1AT significantly attenuated this increase at 3 h (**a**) and 6 h (**b**) stimulation time. Addition of MCC950 did not alter NLRP3-protein levels in all treatment groups. Treatment with Aβ_1–42_ significantly increased mRNA levels of NLRP3 in astrocytes at 3 h (**c**) and 6 h (**d**). Co-treatment with A1AT significantly blocked this increase in NLRP3 mRNA expression. MCC950-pretreatment had no effects on NLRP3 mRNA expression at 3 h and 6 h. Data of *n* = 3 in triplicate represent mean ± SD. *^/a^*p* < 0.05; **^/aa^*p* < 0.01; ^***/aaa^*p* < 0.001, ns not significant compared to untreated cell control (ctr)

We next evaluated the expression of NLRP3 in the given experimental settings. Stimulation of astrocytes with Aβ_1–42_-oligomers significantly increased NLRP3 mRNA-expression by three-fold (Aβ_1–42_, 4 μM) or four-fold (Aβ_1–42_, 10 μM) in comparison to untreated controls (Fig. 3a, Fig. 4c, d). Co-treatment with 4 mg/mL A1AT almost completely blocked this increase (Fig. 3a, Fig. 4c, d). Western blot analysis revealed a significant higher protein expression of NLRP3 in Aβ_1–42_-stimulated astrocytes compared to untreated controls (Fig. 3b, c, Fig. 4a, b). Co-treatment with A1AT significantly prevented this increase in NLRP3 protein expression (Fig. 3c, Fig. 4a, b). These results were confirmed by ICC. Staining with GFAP-, NLRP3-antibody and Hoechst revealed significantly higher fluorescence intensity of NLRP3-stained astrocytes stimulated with 10 μM of Aβ_1–42_ (Fig. 3d, e, microscope settings were identical for each experiment). Fluorescence intensity of Aβ_1–42_-stimulated cells significantly declined with co-treatment by A1AT (Fig. 3e).

To exclude that NLRP3-upregulation was due to microglia contamination, ICC-staining using Iba1- and NLRP3-antibody was performed (Additional file 5: Figure S5). Indeed, NLRP3 was expressed by the few present microglia. But as Additional file 1: Figure S2 and Additional file 5: Figure S5 show, the amount of microglia was so low, that their impact on NLRP3-expression is negligible.

### Aβ_1–42_-stimulation and A1AT-treatment did not regulate ASC expression

**Fig. 5:**
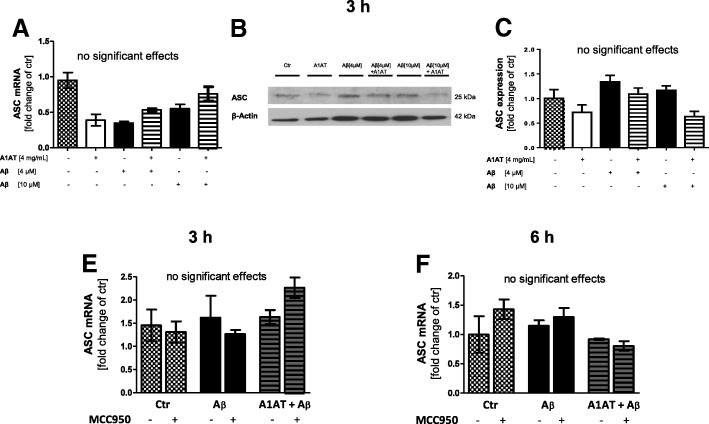
ASC was not regulated by Aβ_1–42_ and A1AT at 3 h and 6 h stimulation. (**a**) Quantitative RT-PCR demonstrated ASC was not regulated by Aβ_1–42_ or A1AT. Data of *n* = 6 in triplicate represent mean ± SD. (**b**–**c**) Western blot revealed no regulation of ASC by Aβ_1–42_ or A1AT. Data of *n* = 3 in triplicate represent mean ± SD. (**e–f**) RT-PCR demonstrated that also at 6 h stimulation time such as MCC950-pretreatment did not regulate ASC mRNA expression. Data of *n* = 3 in triplicate represent mean ± SD. *^/a^*p* < 0.05; **^/aa^*p* < 0.01; ^***/aaa^*p* < 0.001, ns not significant compared to untreated cell control (ctr)

The NLRP3-inflammasome consists of active NLRP3 (LRR, NACHT, PYD, CARD) as well as the adaptor protein ASC and pro-caspase 1. In primary astrocytes, treatment with 4 μM or 10 μM Aβ_1–42_ or 4 mg/mL A1AT did not result in changes of ASC mRNA or protein expression at 3 h and 6 h stimulation time (Fig. 5).

### A1AT abrogated Aβ_1–42_-induced upregulation of caspase 1 and the pro-inflammatory cytokine IL-1β

**Fig. 6:**
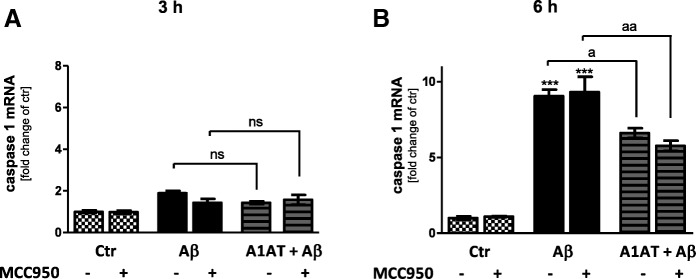
A1AT time-dependently blocked Aβ_1–42_-induced upregulation of caspase 1 mRNA in primary astrocytes. (**b–c**) As quantified by RT-PCR treatment with 4 μM Aβ_1–42_ significantly increased mRNA levels of caspase 1 in astrocytes at 6 h, but not at 3 h. Co-treatment with A1AT blocked this increase in caspase 1 mRNA expression significantly at 6 h, but not at 3 h. Addition of MCC950 had no effects on caspase 1 mRNA expression at both stimulation times. Data of *n* = 3 in triplicate represent mean ± SD.*^/a^*p* < 0.05; **^/aa^*p* < 0.01; ^***/aaa^*p* < 0.001, ns not significant compared to untreated cell control (ctr)

Next, we analyzed caspase 1, IL-1β and IL-18 expression. Whereas 3 h treatment of primary astrocytes with 4 μM of Aβ_1–42_ did not result in an increase of caspase 1 mRNA expression, treatment with 10 μM of Aβ_1–42_ led to a significant increase of caspase 1 mRNA expression (Fig. 6a, Additional file 6: Figure S6A). Co-treatment with 4 mg/mL A1AT blocked the increase of caspase 1 expression significantly (Additional file 6: Figure S6A). Repeating this experiment, this time performing a 6-h stimulation, revealed that Aβ_1–42_ significantly increased mRNA levels of caspase 1 in astrocytes (Fig. 6b). Co-treatment with A1AT blocked this increase in caspase 1 mRNA (Fig. 6b).

**Fig. 7:**
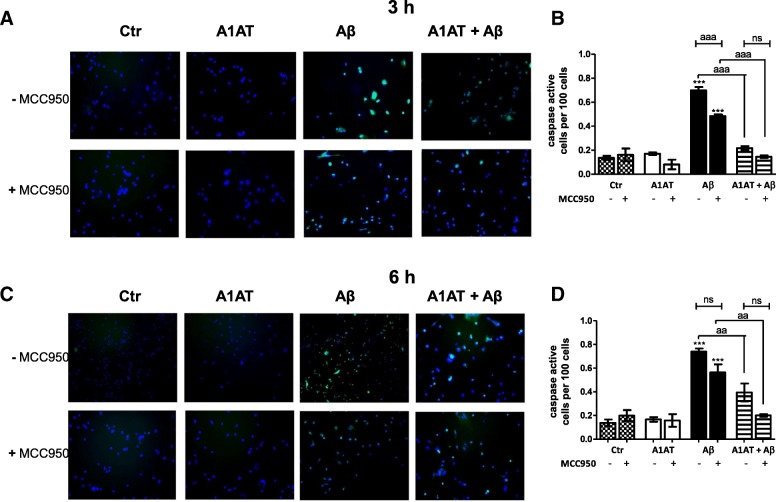
A1AT mitigated caspase activity in Aβ_1–42_-stimulated astrocytes. (**a**, **c**) For convenience, we have only illustrated the overlay images of all treatment groups ± MCC950. Counting of caspase active cells (**b**, **d**) revealed a significant increase of caspase active cells with 4 μM Aβ_1–42_-stimulation at both stimulation times. Co-treatment with A1AT significantly blocked this effect at both stimulation times. In MCC950-treated and Aβ_1–42_-stimulated cells caspase activity significantly declined at 3 h. Additive treatment by MCC950 to A1AT and Aβ_1–42_-stimulated astrocytes did not significantly change caspase activity. Data of *n* = 3 in triplicate represent mean ± SD. *^/a^*p* < 0.05; **^/aa^*p* < 0.01; ^***/aaa^*p* < 0.001, ns not significant compared to untreated cell control (ctr)

FAM-FLICA® Caspase-1 Assay, measuring active caspase-1 enzyme, showed that treatment with Aβ_1–42_ significantly induced the number of caspase 1 positive cells (Fig. 7). Co-treatment with 4 mg/mL of A1AT significantly blocked this increase of caspase 1-positive cells (Fig. 7).

**Fig. 8:**
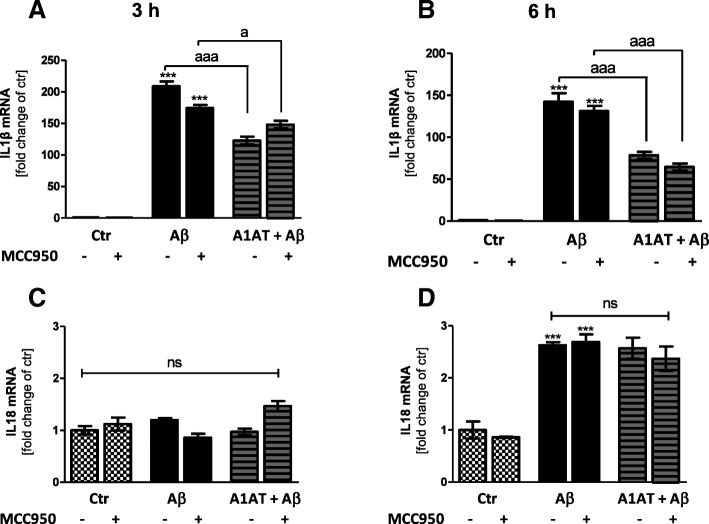
A1AT mitigated Aβ_1–42_-induced upregulation of IL-1β mRNA in primary astrocytes. (**a**–**b**) As quantified by RT-PCR stimulation with 4 μM Aβ_1–42_ significantly increased mRNA levels of IL-1β in astrocytes at 3 h and 6 h. Co-treatment with A1AT blocked this increase significantly at 3 h and 6 h. Addition of MCC950 had no significant impact on gene expression at 3 h and 6 h. (**c**) Aβ_1–42_-stimulation such as A1AT-treatment did not affect IL-18 mRNA expression at 3 h. (**d**) Stimulation with Aβ_1–42_ significantly increased mRNA levels of IL-18 in astrocytes at 6 h. Co-treatment with A1AT did not block this increase. MCC950 had no effects on gene expression of IL-18 at 3 h and 6 h. Data of *n* = 3 in triplicate represent mean ± SD. *^/a^*p* < 0.05; **^/aa^*p* < 0.01; ^***/aaa^*p* < 0.001, ns not significant compared to untreated cell control (ctr)

In the next step, pro-inflammatory cytokines cleaved by caspase 1 were assessed. Stimulation of astrocytes with 4 μM and 10 μM of Aβ_1–42_ increased mRNA expression of IL-1β (Fig. 8a, b, Additional file 6: Figure S6B). Co-treatment with A1AT significantly reduced mRNA expression of IL-1β (Fig. 8a, b, Additional file 6: Fig. 6b). In contrast, IL-18 gene expression was not affected by either of the treatments at 3 h stimulation time (Fig. 8c, Additional file 6: Figure S6C). However, 6 h stimulation with Aβ_1–42_ significantly increased mRNA levels of IL-18 in astrocytes, but A1AT did not block this effect (Fig. 8d).

**Fig. 9:**
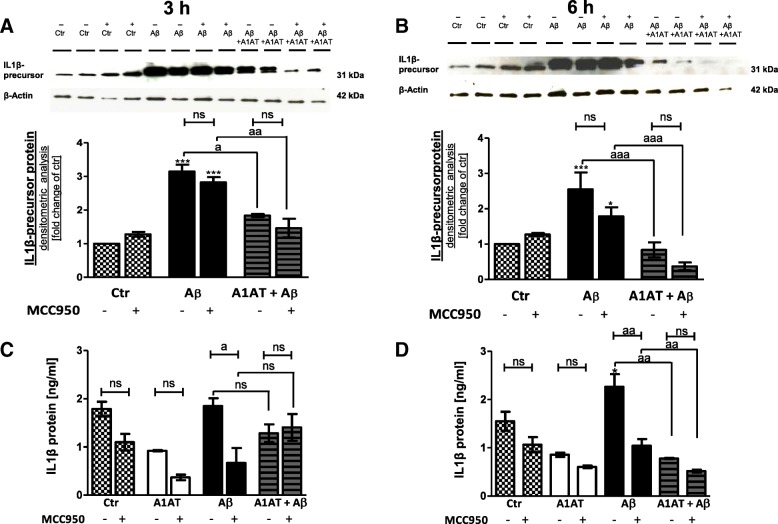
Treatment with A1AT time-dependently mitigated Aβ_1–42_-induced upregulation of IL1β-precursor protein in primary astrocytes. Western blot confirmed an Aβ_1–42_-induced significant increase of of IL1β-precursor protein at 3 h (**a**) and 6 h (**b**). Co-treatment with A1AT significantly attenuated this increase at 3 h and 6 h stimulation time. Addition of MCC950 did not alter levels of IL1β-precursor protein in Aβ_1–42_-stimulated astrocytes. A1AT + in Aβ_1–42_ in the presence of MCC950 did not alter IL1β-precursor protein levels at both 3 h and 6 h. (**c**–**d**) IL-1β-ELISA revealed a time-dependent upregulation of IL-1β protein levels in Aβ_1–42_-stimulated astrocytes, significant at 6 h stimulation. Co-treatment with A1AT significantly decreased protein expression of IL-1β time-dependently, at 6 h stimulation time. The presence of MCC950 in Aβ_1–42_-stimulated decreased IL-1β-expression significantly at both 3 h and 6 h. Further, there is a trend towards a decrease of IL-1β-protein levels in the presence of MCC950 in nearly all treatment groups, though not significant. Co-stimulation of Aβ_1–42_ and A1AT in MCC950-pretreated astrocytes did not affect IL-1β-expression. Data of *n* = 3 in triplicate represent mean ± SD. *^/a^*p* < 0.05; **^/aa^*p* < 0.01; ^***/aaa^*p* < 0.001, ns not significant compared to untreated cell control (ctr)

Furthermore, western blot revealed a significant upregulation of the IL-1β-precursor in Aβ_1–42_-treated cells (Fig. 9a, b). Co-treatment with A1AT blocked the effect (Fig. 9a, b). Analysis of IL-1β protein by ELISA revealed a similar effect of increased levels after Aβ_1–42_-stimulation, which was significant at 6 h, but not at 3 h (9C-D). A1AT-treatment significantly prevented this increase time-dependently after 6 h stimulation (Fig. 9d).

### MCC950 reduced caspase 1 activity and IL-1β protein expression

In order to elucidate a direct interaction between A1AT and the NLRP3-inflammasome, all studies were repeated including MCC950—a selective inhibitor of NLRP3. MCC950 had no impact on cell viability (Fig. 2a, c), but led to an increase of cell metabolism in each treatment group at 3 h and 6 h, though not significant (Fig. 2b, d). In western blot, MCC950 did not change protein levels of NLRP3 (Fig. 4a, b). Treatment with MCC950 did not alter gene expression of NLRP3, ASC, caspase 1, IL-1β, and IL-18 (Figs. 4c, d, 5E–F, 6a, b, 8a, d).

MCC950 significantly mitigated caspase 1 activity (Fig. 7) and significantly decreased IL-1β in Aβ-stimulated astrocytes examined by ELISA (Fig. 9c, d). Further, there is a trend towards a decrease of IL-1β-protein levels in the presence of MCC950 in nearly all treatment groups, though not significant (Fig. 9c, d). MCC950 did not affect Aβ-induced expression of IL-1β-precursor protein in western blot (Fig. 9a, b).

### Co-treatment with A1AT and Aβ in the presence of MCC950 did not alter expression of NLRP3-inflammasome components

Examined by caspase 1 assay (Fig. 7), IL-1β western blot (Fig. 9a, b) and IL-1β-ELISA (Fig. 9c, d) co-treatment with A1AT and Aβ in the presence of MCC950 did not alter expression of inflammasome components compared to the same stimulation group in absence of MCC950. Therefore, we conclude that A1AT mitigated IL-1β mainly by inhibiting NLRP3-inflammasome.

## Discussion

Activation of glia cells and overexpression of pro-inflammatory cytokines are regarded early events in Alzheimer’s disease [56]. In recent years, astrocytes have come into focus in neurodegenerative disorders such as AD and are seen in a new way. Astrocytes express a plethora of receptors and modulate cells and their function in their surroundings [57]. In brief, they are involved in excitotoxic glutamate release, secretion of pro-inflammatory cytokines, growth factor production, stabilization, and organization of the blood-brain barrier and Aβ_1–42_ production [58]. In microglia cells, the activation of NLRP3 appears to be an essential step during AD. Following activation of NLRP3, inflammation is triggered by the activation of caspase 1 and generation of IL-1β. This hampers the phagocytic capability of microglia cells [39]. The NLRP3-inflammasome is important for the initiation and processing of neuroinflammatory processes, and especially in AD, NLRP3 is associated with age-related inflammation [59]. NLRP3 is known to be activated by Aβ_1–42_-aggregates [14]. NLRP3 knock-out in transgenic animals carrying mutations associated with AD prevents AD pathology [24].

Our group has recently presented data in acute and chronic neurodegenerative disease models that different components of the NLRP3-inflammasome are allocated to astrocytes [60–64]. With respect to AD, Couturier and co-workers were able to show that astrocytes produce and release IL-1β following Aβ_1–42_-stimulation [18]. In this animal model, the downregulation of NLRP3-inflammasome activation leads to decreased amyloid plaques and a better memory performance [35]. Our data now show that stimulation of primary astrocytes with Aβ_1–42_ induces a dose-dependent upregulation of NLRP3. This in turn is known to stimulate the activation of caspase 1 and IL-1β [39]. Our work further demonstrates that such an effect also occurs after short-term stimulation with Aβ_1–42_. In contrast, the co-treatment of astrocytes with Aβ_1–42_ and A1AT blocks the induction of NLRP3. The regulation of NLRP3 is complex and usually requires a two-step activation process [22]. Currently, it is well accepted that the first step includes a priming signal, usually provided by NF-ΚB signaling or secretion of endogenous cytokines such as IL-1a [65]. NLRP3 is then activated by a variety of cellular signals, amongst them are misfolded proteins. The large variety of possible regulatory signals and pathways suggest that the activation is rather the consequence of a disturbance of cellular equilibrium [22]. In 2012, Lee and co-workers have identified calcium signaling as essential in NLRP3 activation [66]. In this study, increased intracellular calcium levels are associated with NLRP3 activation. In astrocytes, Aβ_1–42_ potentiates calcium signaling which is triggered by mGlu, α7nAChR, and purinergic substances [57].

Yet, there is little evidence on the regulation of the NLRP3-inflammasome by A1AT. Toldo et al. 2011 stated that A1AT inhibits caspase-1 [67]. Aggarwal et al. 2016 found that—by the presence of polyunsaturated fatty acids—A1AT downregulates NLRP3 and caspase 1 [68]. For astrocytes, no data exist with respect to mechanisms of action of A1AT. In previous studies, we have presented data which show that A1AT reduces inflammation in microglia cells mainly by controlling calcium signaling pathways [15]. A1AT has no effect on classic signaling pathways such as MAPK p38, p44/42, JNK, and cAMP-coupled mechanisms [15]. Using a fluorescent calcium dye, we have shown that A1AT reduces intracellular calcium concentrations in a microglial cell line [15]. A1AT had no direct effect on Aβ_1–42_-oligomerization [15]. We therefore hypothesize that A1AT effects on NLRP3 upregulation in primary astrocytes are mainly triggered by an inhibition of calcium and calpain. This hypothesis needs further evaluation, since other reports also demonstrate that A1AT is able to reduce glutamate-induced toxicity in murine primary neurons [45]. Since astrocytes release glutamate in response to Aβ_1–42_-stimulation, this could represent another way how A1AT prevents deleterious Aβ_1–42_-induced inflammatory cascades in microglia, neurons, and astrocytes. However, our current study does not include research on how A1AT could regulate the NLRP3-inflammasome complex. Further, it must be remarked that microglia might have partially contributed to the observed results due to the slight contamination of approximately 5%.

Our data demonstrate that NLRP3-inflammasome components are upregulated time-dependently following Aβ_1–42_-stimulation. This can be blocked by A1AT application. NLRP3 is the sensor protein of the NLRP3-inflammasome complex, which explains its upregulation after Aβ_1–42_-stimulation. ASC, in contrast, has the caspase activating and recruitment domain. In our experiments, ASC concentration on the mRNA and protein level was unchanged, indicating that Aβ_1–42_ and A1AT mainly regulate NLRP3, but not ASC. We assume the total amount of ASC to be sufficient to lead to the NLRP3-ASC-complex formation and caspase 1 binding.

To further investigate the direct effect of A1AT on inflammasome-dependent IL-1β maturation, we used a specific NLRP3-inhibitor called MCC950. As previously shown by Coll et al., pre-treatment with MCC950 prevents complex formation of apoptosis-associated speck-like protein containing a CARD (ASC) and blocks the release of IL-1β in immunological active cells, without affecting priming of NLRP3 [46]. Furthermore, MCC950 stimulated Aβ phagocytosis in vitro, and it reduced Aβ accumulation in a mouse model of AD, which was associated with improved cognitive function [50]. In our studies, MCC950-pretreatment in cells co-treated with A1AT and Aβ did not further drop IL-1β protein expression. Thus, A1AT had no effect on protein expression, when NLRP3 was selectively blocked. We therefore conclude that A1AT reduces IL-1β by inhibiting NLRP3-inflammasome.

These observations not only clearly highlight the importance of astroglial-related pro-inflammatory processes in the brain and in particular during AD, but also point at A1AT as a potent antagonist in astrocyte-dependent inflammatory signaling.

## Conclusion

We demonstrate that Aβ_1–42_-stimulation results in an upregulation of NLRP3, caspase 1, and its cleavage products in astrocytes. A1AT time-dependently hampers Aβ_1–42_-triggered neuroinflammation by attenuating NLRP3-inflammasome expression. This suggests that A1AT offers a therapeutic opportunity for AD treatment.

## Additional files

Additional file 1: **Figure S2.** Cell counting revealed 95.2% astrocytes and 4.5% microglia. Approximately 0.4% of the cells remained undefined. *n* = 12. (PDF 336 kb)

Additional file 2: **Figure S1.** There are no oligodendrocytes contaminating the cell culture. Non-specific binding of Olig2 on astrocytes was observed. (PDF 316 kb)

Additional file 3: **Figure S3.** (A) Stimulation with Aβ_1–42_ led to a concentration-dependent LDH-release. For further experiments, Aβ_1–42_ (10 μM) was selected as the maximum concentration to not exceed 50% of cell death. (B) Ascending concentrations of A1AT did not affect cell viability. (C) Co-treatment with Aβ_1–42_ and A1AT did not affect LDH-release, whereas LPS significantly increased LDH-release. Treatment with A1AT significantly reduced LDH-release in LPS-stimulated astrocytes. Data of *n* = 6 in triplicate represent mean ± SD. *^/a^*p* < 0.05; **^/aa^*p* < 0.01; ^***/aaa^*p* < 0.001, ns not significant compared control. (PDF 246 kb)

Additional file 4: **Figure S4.** No significant differences in cell metabolism were detected after increasing concentrations of Aβ_1–42_ (A), A1AT (B) or co-treatment of A1AT, Aβ_1–42_ and LPS (C). Data of *n* = 6 in triplicate represent mean ± SD, *ns* not significant compared to control. (PDF 260 kb)

Additional file 5: **Figure S5.** (A) Since there are no oligodendrocytes contaminating the cell culture, NLRP3-expression was not oligodendrocyte-induced. (B) NLRP3-expression was indeed induced by the few present microglia. But the majority of NLRP3-expression was not microglia-mediated. *n* = 3. (PDF 385 kb)

Additional file 6: **Figure S6.** (A) 10 μM Aβ_1–42_ significantly increased caspase 1 mRNA. Co-treatment with A1AT blocked the increase of caspase 1 mRNA expression significantly. (B) Stimulation with Aβ_1–42_ significantly increased IL-1β mRNA. Gene expression of IL-1β was significantly reduced with A1AT-co-treatment. (C) In contrast, Aβ_1–42_-stimulation such as A1AT-treatment did not affect IL-18 mRNA expression. Data of *n* = 6 in triplicate represent mean ± SD. *^/a^*p* < 0.05; **^/aa^*p* < 0.01; ^***/aaa^*p* < 0.001 compared to control. (PDF 238 kb)

## Abbreviations

A1AT: α1-antitrypsin
AD: Alzheimer’s disease
ASC: Apoptosis-associated speck-like protein containing a CARD
Aβ: Amyloid β
CARD: Caspase activation and recruitment domain
Casp1: Caspase 1
CNS: Central nervous system
Ctr: Control
DMSO: Dimethylsulfoxid
GAPDH: Glycerinaldehyd-3-phosphate-dehydrogenase
GFAP: Glial fibrillary acidic protein
HPRT: Hypoxanthine phosphoribosyltransferase
Hsp90: Heat shock protein 90
Iba1: Ionized calcium binding adaptor molecule 1
ICC: Immunocytochemistry
IL-18: Interleukin-18
IL-1β: Interleukin-1β
LDH: Lactate dehydrogenase
LPS: Lipopolysaccharide
LRR: Leucine-rich repeat
NACHT: NAIP (NLP family apoptosis inhibitor protein), CIITA (class 2 transcription activator), HET-E (heterokaryon incompatibility), TEP1 (telomerase-associated protein 1)
NLRP: NACHT, LRR, and PYD domains-containing
PCR: Polymerase chain reaction
Pos: Positive control
PYD: Pyrin domain
q-RT-PCR: Quantitative real-time PCR
RFU: Relative fluorescence units
sq-PCR: Semi-quantitative PCR
