# Supplementary figures and images for: α1-antitrypsin mitigates NLRP3-inflammasome activation in amyloid β1–42-stimulated murine astrocytes
Source: J Neuroinflammation. 2018 Sep 27;15:282. doi: 10.1186/s12974-018-1319-x (PMC6158809; doi:10.1186/s12974-018-1319-x)

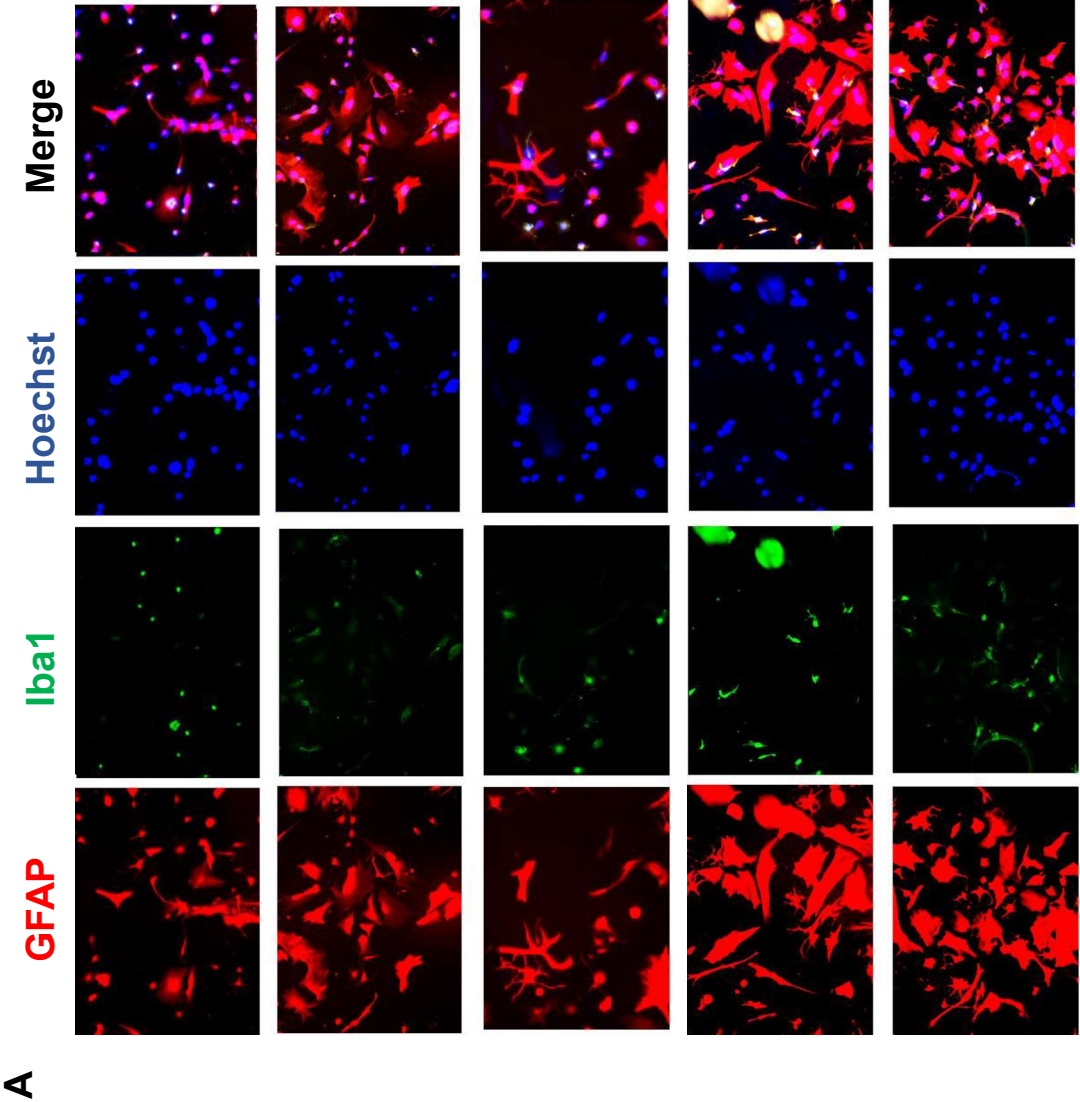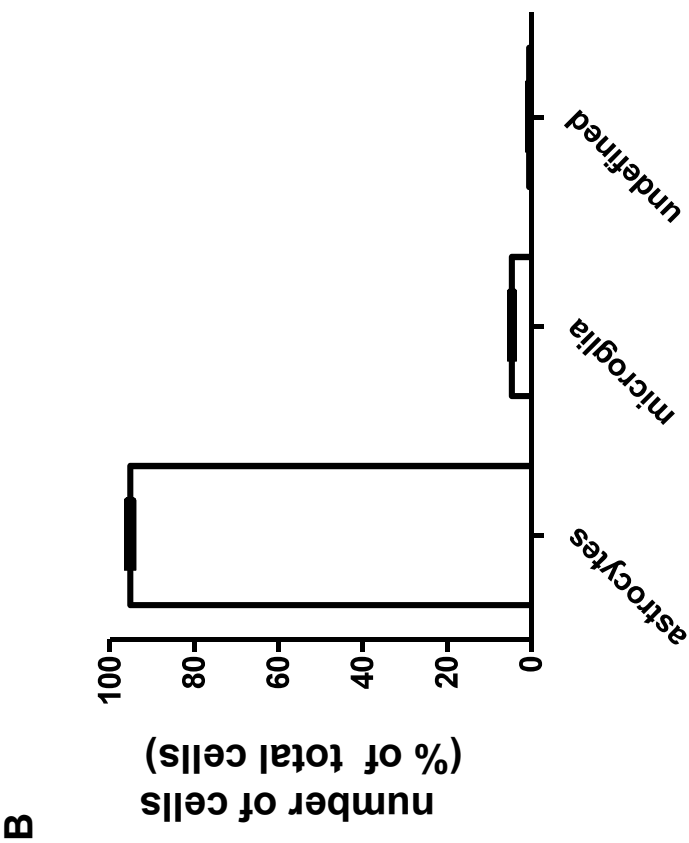

Supplement: Supplementary file 1 — Figure S2. Cell counting revealed 95.2% astrocytes and 4.5% microglia. Approximately 0.4% of the cells remained undefined. n = 12. (PDF 336 kb) [file 12974_2018_1319_MOESM1_ESM.pdf]

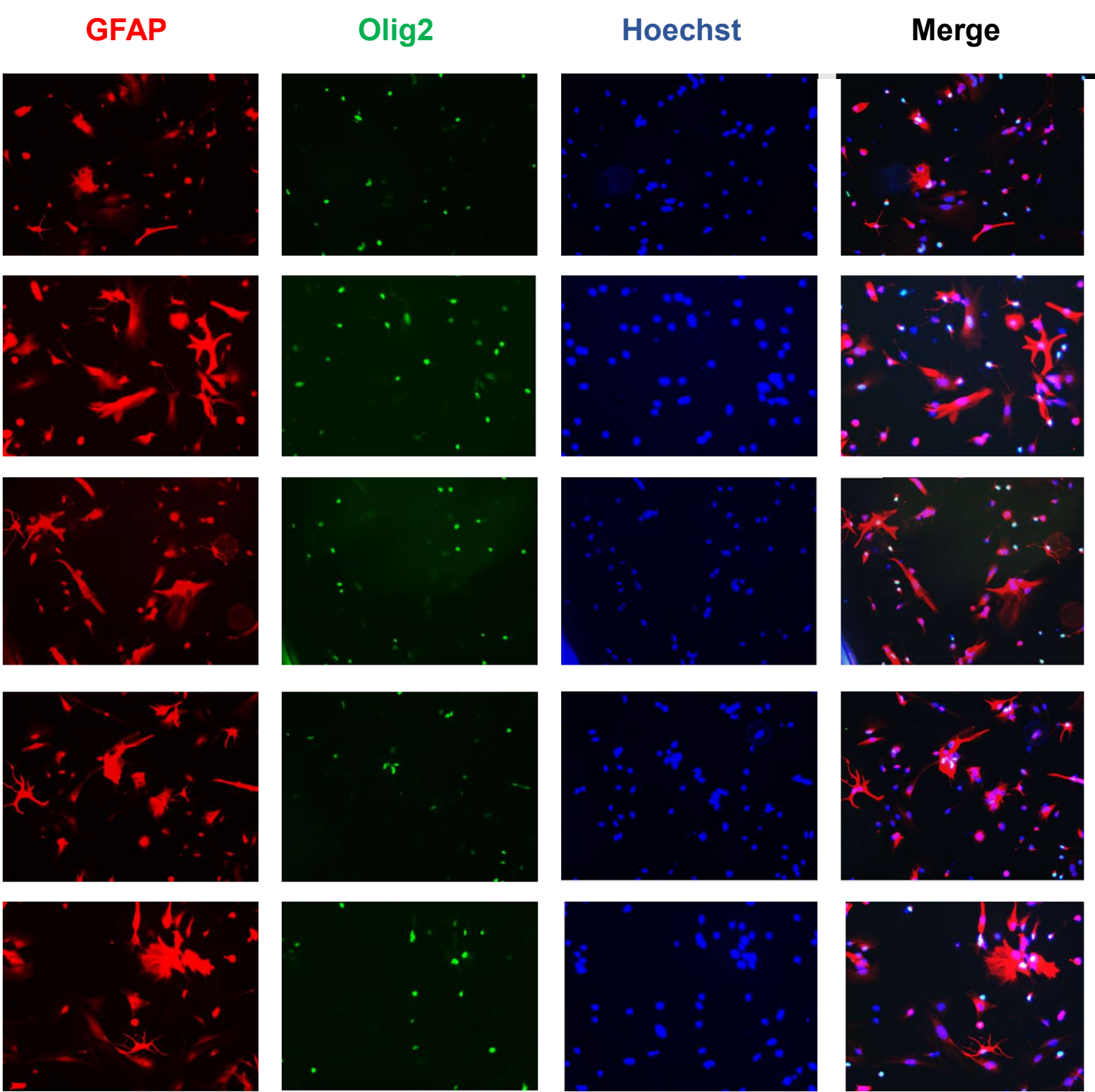

Supplement: Supplementary file 2 — Figure S1. There are no oligodendrocytes contaminating the cell culture. Non-specific binding of Olig2 on astrocytes was observed. (PDF 316 kb) [file 12974_2018_1319_MOESM2_ESM.pdf]

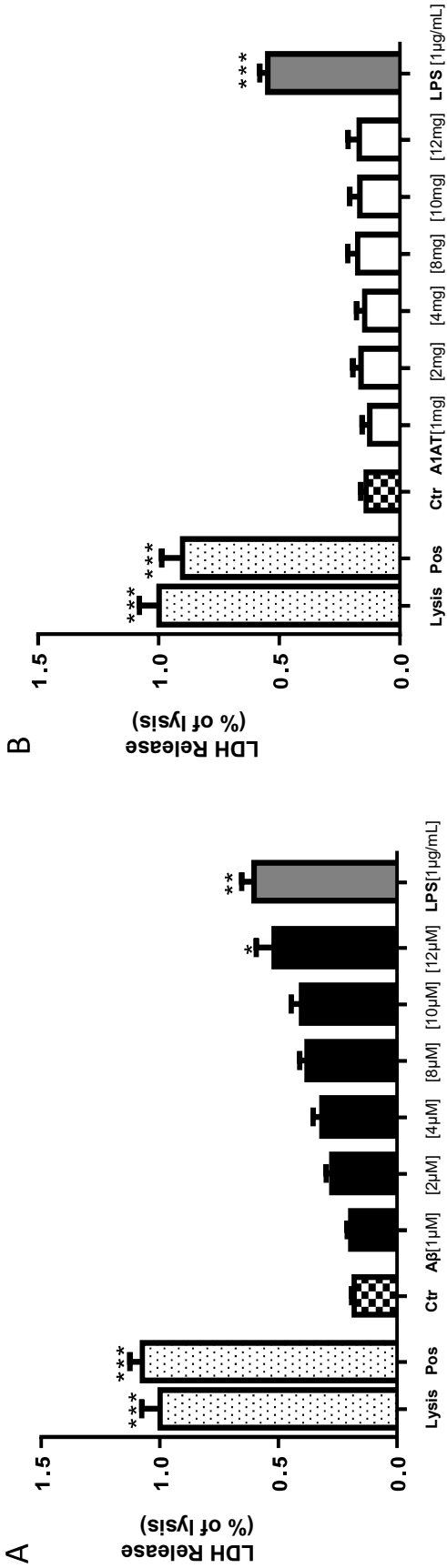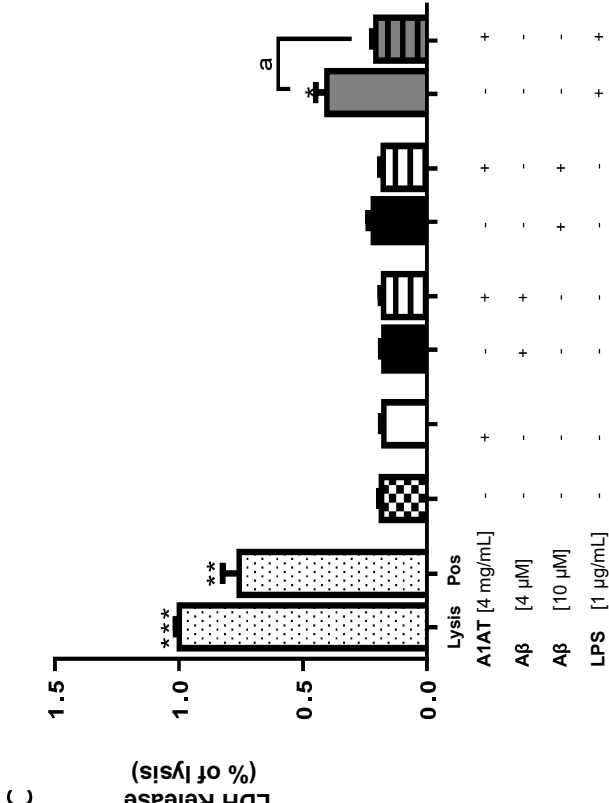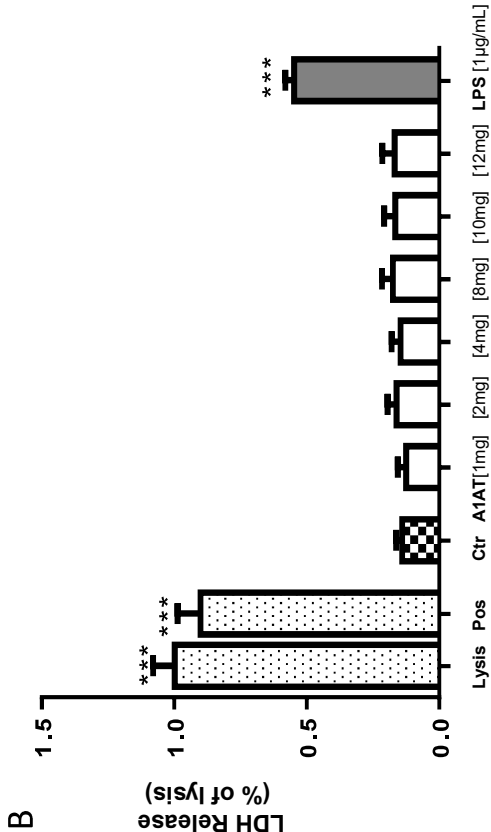

Supplement: Supplementary file 3 — Figure S3. (A) Stimulation with Aβ1–42 led to a concentration-dependent LDH-release. For further experiments, Aβ1–42 (10 μM) was selected as the maximum concentration to not exceed 50% of cell death. (B) Ascending concentrations of A1AT did not affect cell viability. (C) Co-treatment with Aβ1–42 and A1AT did not affect LDH-release, whereas LPS significantly increased LDH-release. Treatment with A1AT significantly reduced LDH-release in LPS-stimulated astrocytes. Data of n = 6 in triplicate represent mean ± SD. */ap < 0.05; **/aap < 0.01; ***/aaap < 0.001, ns not significant compared control. (PDF 246 kb) [file 12974_2018_1319_MOESM3_ESM.pdf]

A

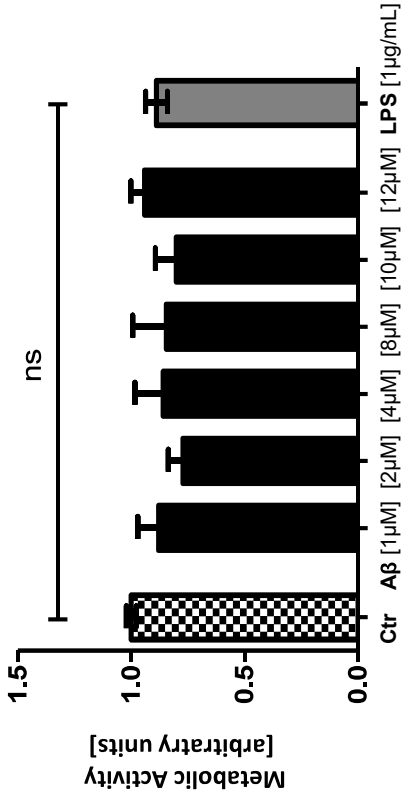

B

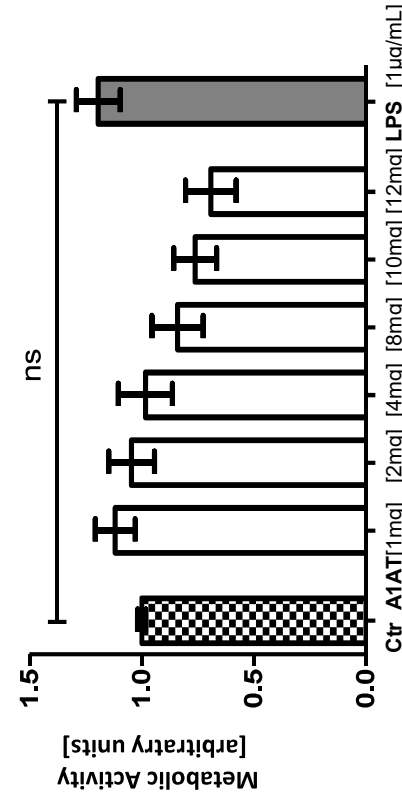

C

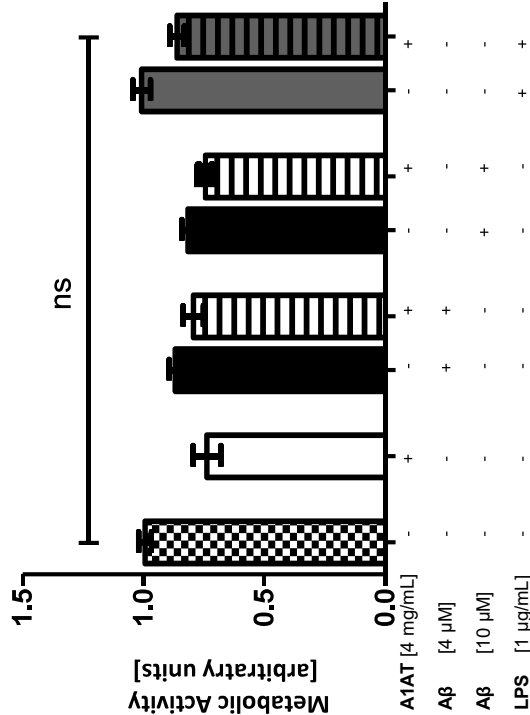

Supplement: Supplementary file 4 — Figure S4. No significant differences in cell metabolism were detected after increasing concentrations of Aβ1–42 (A), A1AT (B) or co-treatment of A1AT, Aβ1–42 and LPS (C). Data of n = 6 in triplicate represent mean ± SD, ns not significant compared to control. (PDF 260 kb) [file 12974_2018_1319_MOESM4_ESM.pdf]

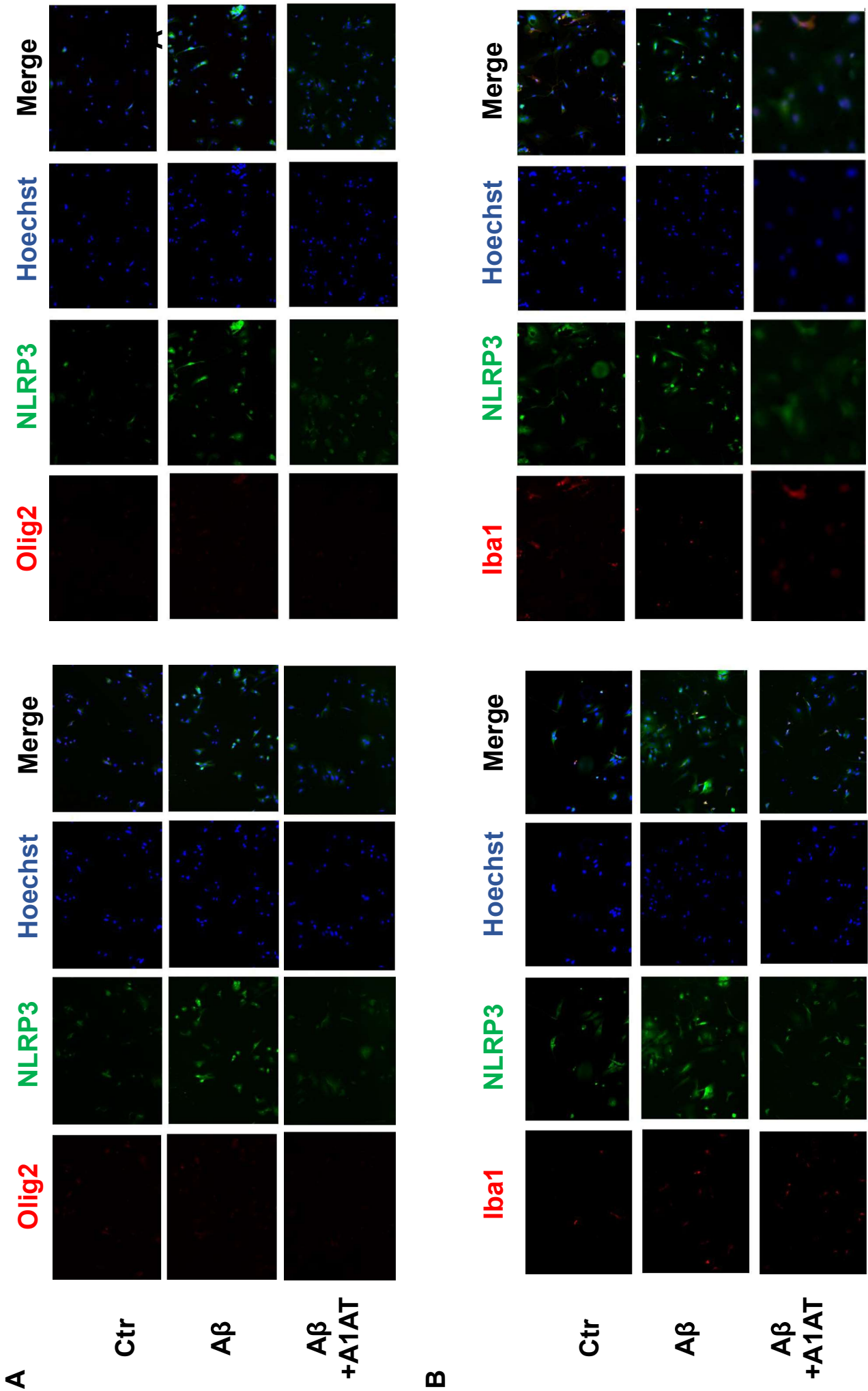

Supplement: Supplementary file 5 — Figure S5. (A) Since there are no oligodendrocytes contaminating the cell culture, NLRP3-expression was not oligodendrocyte-induced. (B) NLRP3-expression was indeed induced by the few present microglia. But the majority of NLRP3-expression was not microglia-mediated. n = 3. (PDF 385 kb) [file 12974_2018_1319_MOESM5_ESM.pdf]

A

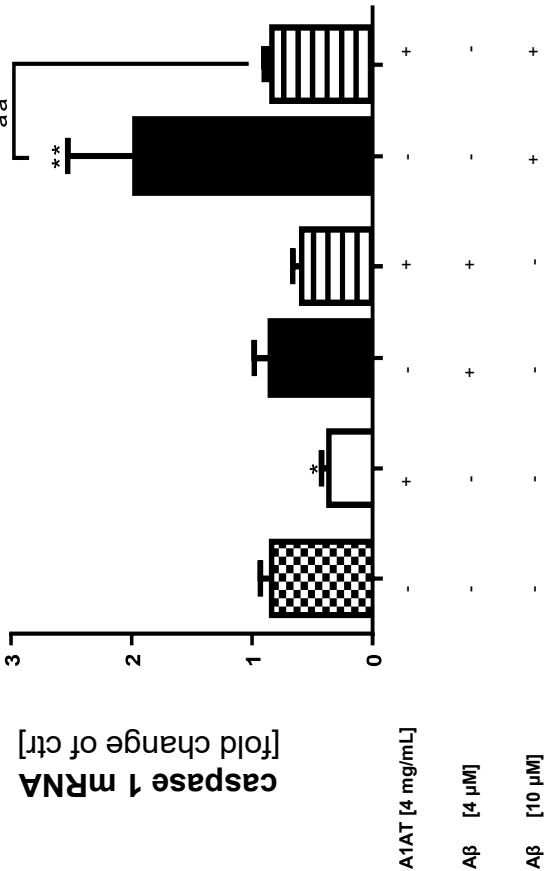

B

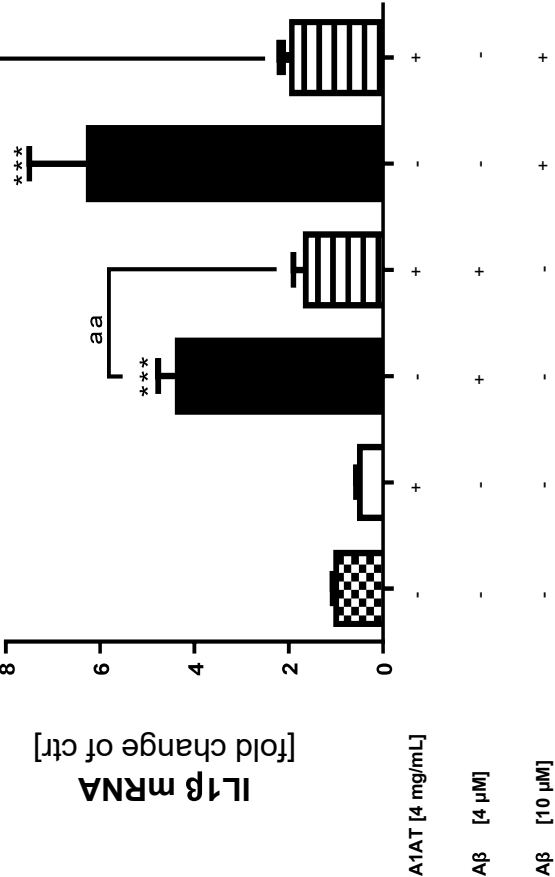

C

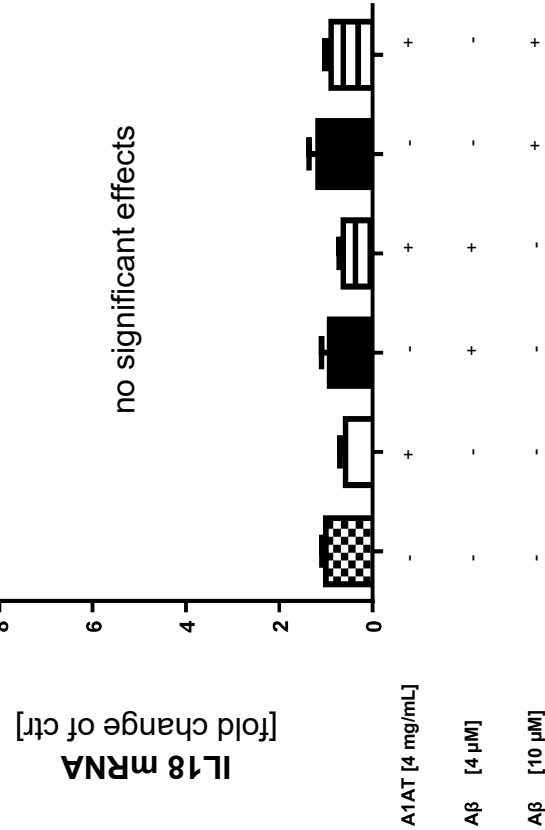

Supplement: Supplementary file 6 — Figure S6. (A) 10 μM Aβ1–42 significantly increased caspase 1 mRNA. Co-treatment with A1AT blocked the increase of caspase 1 mRNA expression significantly. (B) Stimulation with Aβ1–42 significantly increased IL-1β mRNA. Gene expression of IL-1β was significantly reduced with A1AT-co-treatment. (C) In contrast, Aβ1–42-stimulation such as A1AT-treatment did not affect IL-18 mRNA expression. Data of n = 6 in triplicate represent mean ± SD. */ap < 0.05; **/aap < 0.01; ***/aaap < 0.001 compared to control. (PDF 238 kb) [file 12974_2018_1319_MOESM6_ESM.pdf]
